# Polycistronic gene expression in *Aspergillus niger*

**DOI:** 10.1186/s12934-017-0780-z

**Published:** 2017-09-25

**Authors:** Tabea Schuetze, Vera Meyer

**Affiliations:** Department of Applied Microbiology and Genetics, Technische Universität Berlin, Gustav-Meyer-Allee 25, 13355 Berlin, Germany

**Keywords:** P2A peptide, *Aspergillus niger*, Luciferase, Enniatin B, Polycistronic, Heterologous gene expression, Secondary metabolite

## Abstract

**Background:**

Genome mining approaches predict dozens of biosynthetic gene clusters in each of the filamentous fungal genomes sequenced so far. However, the majority of these gene clusters still remain cryptic because they are not expressed in their natural host. Simultaneous expression of all genes belonging to a biosynthetic pathway in a heterologous host is one approach to activate biosynthetic gene clusters and to screen the metabolites produced for bioactivities. Polycistronic expression of all pathway genes under control of a single and tunable promoter would be the method of choice, as this does not only simplify cloning procedures, but also offers control on timing and strength of expression. However, polycistronic gene expression is a feature not commonly found in eukaryotic host systems, such as *Aspergillus niger*.

**Results:**

In this study, we tested the suitability of the viral P2A peptide for co-expression of three genes in *A. niger*. Two genes descend from *Fusarium oxysporum* and are essential to produce the secondary metabolite enniatin (*esyn1*, *ekivR*). The third gene (*luc*) encodes the reporter luciferase which was included to study position effects. Expression of the polycistronic gene cassette was put under control of the Tet-On system to ensure tunable gene expression in *A. niger*. In total, three polycistronic expression cassettes which differed in the position of *luc* were constructed and targeted to the *pyrG* locus in *A. niger*. This allowed direct comparison of the luciferase activity based on the position of the luciferase gene. Doxycycline-mediated induction of the Tet-On expression cassettes resulted in the production of one long polycistronic mRNA as proven by Northern analyses, and ensured comparable production of enniatin in all three strains. Notably, gene position within the polycistronic expression cassette matters, as, luciferase activity was lowest at position one and had a comparable activity at positions two and three.

**Conclusions:**

The P2A peptide can be used to express at least three genes polycistronically in *A. niger*. This approach can now be applied to heterologously express entire secondary metabolite gene clusters polycistronically or to co-express any genes of interest in equimolar amounts.

**Electronic supplementary material:**

The online version of this article (doi:10.1186/s12934-017-0780-z) contains supplementary material, which is available to authorized users.

## Background

The filamentous fungus *Aspergillus niger* is one of the main microbial cell factories used in biotechnology for the production of various organic acids, proteins and enzymes [1, 2]. In 2014, *A. niger* was re-engineered to produce the secondary metabolite enniatin heterologously in amounts comparable to the natural producer, *Fusarium oxysporum* was shown [3]. This study not only paved the way for establishing *A. niger* as an industrial producer of homologous or heterologous natural products with pharmaceutical applications, but also enabled studies aiming at overexpression of fungal chimeric enzymes synthesising new-to-nature compounds. This was impossible in bacterial or yeast expression systems due to low expression levels [4]. Given the recent advances in fungal genome editing, sophisticated genetic and metabolic engineering approaches are now feasible for *A. niger*. Various selection markers are available for *A. niger* [5, 6], the split marker approach [7] is commonly used to delete genes, and CRISPR/Cas9 has been experimentally validated in numerous filamentous fungal systems [8, 9]. However, controlled co-expression of a set of genes, or entire pathways, is still technically challenging and has not been shown in *A. niger* to date. In 2014, two publications have reported the successful use of a viral peptide for polycistronic gene expression in fungi. Beekwilder et al. established it for the yeast *Saccharomyces cerevisiae* to produce $\upbeta$-carotene and Unkles et al. were able to express the penicillin gene cluster, encoding the ACV synthetase (pcbAB), isopenicillin N synthase (pcbC) and isopenicillin N acyltransferase (penDE) in the filamentous fungus *Aspergillus nidulans* using the P2A peptide [10, 11]. The use of the viral 2A peptide for polycistronic gene expression in eukaryotes was first described in 2004 [12], where three different 2A sequences (E2A—equine rhinitis A virus, F2A—foot-and-mouth disease virus and T2A—*Thoseaasigna* virus) were used to assemble four transmembrane proteins of the T-cell receptor CD3 complex.

**Fig. 1:**
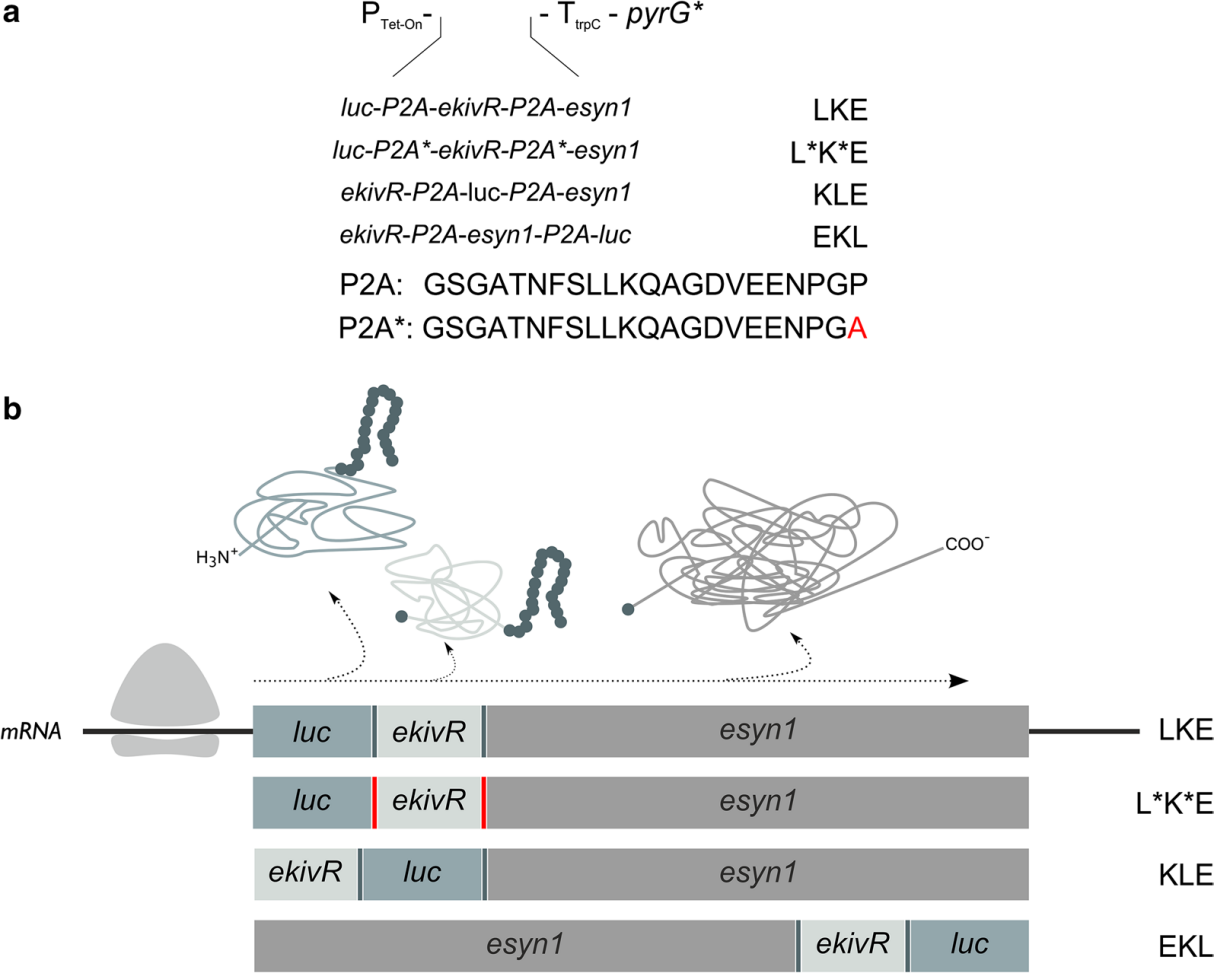
Tet-On based P2A-mediated polycistronic expression system for *A. niger*. **a** Protein sequences of the P2A peptide and the putatively non-functional P2A peptide are given. For the latter, the final C-terminal amino acid proline was exchanged for an alanine. General design of the Tet-On system has been described earlier [26]. Order of genes and abbreviations used for the *A. niger* transformants made are described in the main text. **b** Schematic illustration of the proteins produced by ribosomal translation and position of the 2A added tags at the N- or C-terminal ends of the proteins

The underlying principle of 2A peptide-based co-translation is called the “stop-carry on mechanism”. In brief, the corresponding DNA sequence of an about 20 amino acid long 2A peptide is placed between the genes for co-expression (Fig. 1a). During transcription, a long, polycistronic mRNA is formed, which is used as a template for ribosome-mediated translation. When the ribosome translates the 2A sequence, it skips the formation of a peptide bond between the last two amino acids (glycine, proline) but continues translation. As a result, equimolar amounts of different proteins become translated from a single transcript [13]. Kim et al. analysed the functionality of four different 2A sequences (E2A, F2A, T2A and P2A—Porcine teschovirus) in different organisms and concluded that the P2A peptide has the highest cleavage efficiency [14]. In addition, the system was proven to work in nematodes [15] and *Pichia pastoris*. In the latter system, nine genes were co-expressed from a bidirectional promoter to produce carotenoids and violaceins [16]. Notably, proteins expressed polycistronically by use of any of the 2A peptides become modified, which in turn might impact their activities. They carry an additional proline on their N-terminus and the remaining amino acids of the 2A peptide at their C terminus (Fig. 1b). A potential negative effect of such 2A peptide-mediated protein modification was most recently reported for the filamentous fungus *Trichoderma reesei*. Bicistronic expression of the enhanced green fluorescent protein (eGFP) and a cellobiohydrolase using the F2A peptide allowed detection of a functional eGFP when it was expressed at the second position, but not at the first [17].

In the present study, we aimed to adapt and evaluate the P2A system for *A. niger* by co-expressing three different genes. We expressed luciferase and two genes required for production of the secondary metabolite enniatin (*ekivR*, *esyn1*) polycistronically under control of the inducible Tet-On system. The latter two genes originate from *F. oxysporum*, *esyn1* encodes the nonribosomal peptide synthetase ESYN, and *ekivR* the ketoisovalerate reductase KivR, which is required for synthesis of the precursor molecule d-hydroxyisovalerate [3]. Our results show that polycistronic gene expression did indeed allow expression of all three proteins and the successful production of enniatin. The data furthermore demonstrate that, in the case of luciferase, gene position within the polycistronic construct has important consequences for luciferase activity.

## Results and discussion

### Engineering of strains heterologously expressing polycistronic expression cassettes

In order to test and evaluate the functionality of the P2A peptide in *A. niger* and to study potential position effects within the expression cassette, three different polycistronic gene expression cassettes were designed. By placing the luciferase gene at position one, two and three, and by keeping the order of *ekivR* and *esyn1* constant, the final expression cassettes had the order LKE, KLE and EKL, respectively, whereby ‘L’ stands for luciferase, ‘K’ for ketoisovalerate reductase and ‘E’ for enniatin synthase (Fig. 1). As a negative control for construct LKE, we furthermore designed a fourth polycistronic construct (L*K*E*), in which a putatively non-functional P2A peptide was included. There, the final proline was exchanged for an alanine. In vitro and in vivo data have shown that this leads to a non-functional 2A peptide in *S. cerevisiae* causing translation of a fusion protein [10, 13]. All polycistronic gene cassettes were put under control of the Tet-On system and targeted to the *pyrG* locus of *A. niger*. Details on cloning and selection of *A. niger* transformants each carrying a single copy of the respective plasmid at the *pyrG* locus are given in Additional file 1. Strains that have been selected for further analyses are summarised in Table 1.

**Table 1:** *Aspergillus niger* strains used in this study

Strain name	Relevant genotype/description	Abbreviation	References
AB1.13	*prtT13*, *pyrG378*	R	[22]
ÖV4.10	Tet-On-*esyn1*-T$_{trpC}$, *pyrG* $^+$, single copy; P$_{gpdA}$-*ekivR*-T$_{trpC}$-*amdS*	E + K	[3]
XM1.7	Tet-On-*luc*-T$_{trpC}$, *pyrG* $^+$, single copy	L	[18]
TS38.3	Tet-On-*luc*-*P2A*-*ekivR*-*P2A*-*esyn1*-T$_{trpC}$, *pyrG* $^+$, single copy	LKE	This study
TS39.4	Tet-On-*luc*-*P2A* $^*$-*ekivR*-*P2A* $^*$-*esyn1*-T$_{trpC}$, *pyrG* $^+$, single copy	L*K*E	This study
TS40.4	Tet-On-*ekivR*-*P2A*-*luc*-*P2A*-*esyn1*-T$_{trpC}$, *pyrG* $^+$, single copy	KLE	This study
TS41.13	Tet-On-*ekivR*-*P2A*-*esyn1*-*P2A*-*luc*-T$_{trpC}$, *pyrG* $^+$, single copy	KEL	This study

### Luciferase activity and enniatin B production

**Fig. 2:**
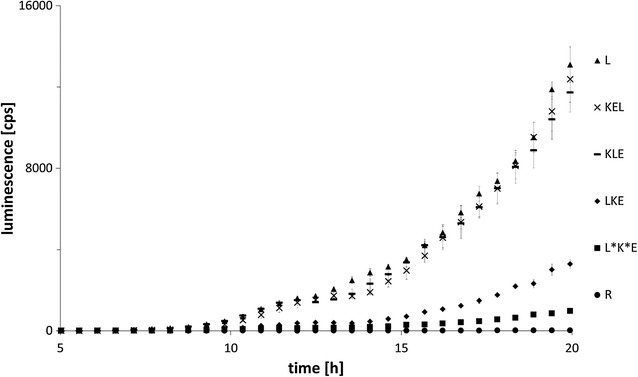
Analysis of reporter activity. Luminescence data of the indicated strains cultivated in microtiter plates and expressing the luciferase gene mono- or polycistronically, n = 3. For experimental details, see “Methods” section

To analyse the luciferase activity, luminescence was measured in *A. niger* transformants which were cultivated in microtiter plates in biological triplicates. In addition to the recipient strain (‘R’) and strains carrying the respective polycistronic construct, a control strain expressing the luciferase gene monocistronically under the Tet-On promoter system and targeted to the *pyrG* locus was included in the analyses (strain ‘L’, [18]). As expected, all strains except for the recipient strain showed an increase in luminescence during the cultivation and only in the presence of the inducing molecule doxycycline (Fig. 2 and data not shown). Surprisingly, luminescence also continuously increased in the L*K*E strain though at very low levels. This might suggest that, in contradiction to data reported for *S. cerevisiae*, the mutated version of P2A is still recognised by ribosomes in *A. niger* but with low efficiency and/or that a long fusion protein has been formed consisting of all three enzymes partly allowing luciferase to be functional. It is well-known that luciferase can function in cis when fused to other proteins [19], hence both events are possible and not mutually exclusive. As depicted in Fig. 2, the strains L, KLE and KEL cluster together according to their luminescence profile in time, whereas strain LKE, where luciferase is placed at position one within the polycistronic cassette, shows only 27% of the luminescence compared to strains KLE and KEL. This observation, indicates on the one hand that an additional proline at the N-terminus is beneficial for luciferase activity, whereas the addition of 21 amino acids to the C-terminus does not interfere with luminescence activity and/or stability. On the other hand, it can be concluded that expression levels and/or enzyme activity of the polycistronic gene cassettes can be as high as the ones from a strain expressing luciferase monocistronically. Notably, the different activities of luciferase at the three positions imply that the P2A-mediated addition of amino acids at the N- and C-terminus of the proteins of interest need to be analysed individually and cannot be predicted. In the case of bicistronic expression of eGFP and cellobiohydrolase in *T. reesei*, eGFP placed at position one (consequently modified at its C-terminus) showed reduced stability and/or fluorescence compared to its placement at position two. No position effects were observed for cellobiohydrolase [17]. Still, these effects do not prevent efficient application of polycistronic gene expression in a host of interest.

The additional C-terminal amino acids could potentially be removed by inclusion of a furin cleavage site. This has been successfully demonstrated in a mammalian cell line (HEK293) producing the heavy and light chain of an antibody using the F2A peptide. A higher antibody concentration was measured in the serum when the cleavage site was present [20].

**Table 2:** Enniatin B production of strains expressing *ekivR* and *esyn1* mono- and polycistronically using 1 and 5% of glucose as carbon source

Glucose concentration (%)	Strain name	Abbreviation	Enniatin B concentration (mg/g dry solids)
1	AB1.13	R	0 ± 0
1	ÖV4.10	K + E	4.72 ± 1.06
1	TS38.3	LKE	1.73 ± 0.52
1	TS39.4	L*K*E	1.14 ± 0.19
1	TS40.4	KLE	2.07 ± 0.33
1	TS41.13	KEL	2.05 ± 0.36
5	AB1.13	R	0 ± 0
5	ÖV4.10	K + E	29.33 ± 1.64
5	TS38.3	LKE	7.81 ± 1.33
5	TS39.4	L*K*E	3.13 ± 0.57
5	TS40.4	KLE	9.19 ± 1.00
5	TS41.13	KEL	15.41 ± 4.07

Experiments were done in biological triplicates. Enniatin was extracted from the freeze-dried solid fraction

Concomitant with luciferase activity, the production of enniatin B was analysed in two different media varying in their glucose concentration. The purpose of the experiment was not to analyse the impact of different positions within the polycistronic cassette, but to provide proof-of-principle that secondary metabolite production is generally possible via P2A-mediated co-translation of the biosynthetic genes *esyn1* and *ekivR* in *A. niger*. As a control, a strain expressing the enniatin synthetase under the Tet-On promoter system and the ketoisovalerate reductase gene constitutively under the *gpdA* promoter was used [3]. The expression and location of the *esyn1* gene in this strain is comparable to the strains with the polycistronic cassettes because it is present as single copy at the *pyrG* locus. However, this *A. niger* strain contains multiple copies of the *ekivR* gene in the genome [3] and is thus likely expressing more KivR enzyme compared to the strains expressing the *ekivR* gene polycistronically. The corresponding results of biological triplicate experiments are summarised in Table 2. At both glucose concentrations all strains except for the recipient strain produced enniatin B. Notably, all strains expressing *ekivR* and *esyn1* polycistronically produced less compared to the strain expressing them monocistronically, in case of 1% glucose, approximately 2.4-fold less. This suggests that the KivR enzyme catalyses the rate-limiting step in the enniatin biosynthesis, a hypothesis which needs to be verified in future experiments. Interestingly, the strain with a putatively non-functional P2A did produce enniatin B as well and concentrations in case of 1% glucose were about 1.5-fold less compared to the strain with a functional P2A (LKE). At 5% glucose, the difference among the strains expressing the genes required for enniatin B production polycistronically was more pronounced (Table 2). Strain KEL produced the most and the putatively non-functional P2A about 2.5-fold less compared to its functional analogue. Again the strain expressing the two genes monocistronically produced approximately 2.7-fold more enniatin B. These data unambiguously show that enniatin B can be produced by expressing genes required for its synthesis polycistronically in *A. niger* and imply that the efficiency of enniatin synthesis might be improved by genetic and/or metabolic engineering approaches of the KivR reaction step. The fact that the putatively non-functional P2A showed luciferase activity as measured by luminescence and enniatin B production indicates that either the fusion protein is active at low efficiency, that the non-functional P2A is processed as well, that proteases might cleave the resulting fusion protein and/or a combination thereof.

### Validation of polycistronic gene expression on mRNA and protein level

**Fig. 3:**
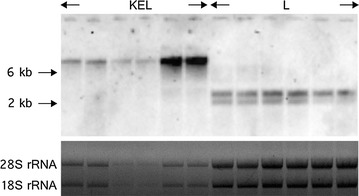
Verification of mono-and polycistronic gene expression using Northern analysis. A fragment of the luciferase gene was used as a probe to detect *luc* mRNA in all strains. Results are exemplarily shown for the strains KEL and L in biological triplicates. Each sample was applied twice. rRNA bands are shown as a loading control. Note that a double band in the L sample is indicative for the two polyadenylation sites present in the *trpC* terminator as described earlier [27]. These two bands are not indistinguishable in the KEL sample due to the large size of the polycistronic mRNA. For details on the abbreviations the reader is referred to Table 1

In order to prove the presence of one long mRNA and single proteins for the polycistronic strains, Northern and Western blot analyses were conducted. One long, mRNA could indeed be detected and differed significantly in size compared to RNA isolated from a strain expressing the luciferase monocistronically (Fig. 3). For Western analyses, a monoclonal antibody against luciferase was used to verify the presence of luciferase and analyse the formation of a fusion protein. As expected, no luciferase signal could be detected for the recipient strain, whereas all other strains showed a signal between 55 and 70 kDa (data not shown) with an expected size of 61 kDa for luciferase. Surprisingly, none of the polycistronic expression strains showed a band above 70 kDa, suggesting high efficiency processing of the P2A peptide. However, in case of the non-functional P2A a fusion protein was expected. Previous reports in *S. cerevisiae* as well as in vitro studies demonstrated that mutation of the final proline to an alanine prevents processing of the 2A peptide, therefore resulting in the production of a fusion protein [10, 13]. In another study, 52 2A mutants were analysed and very little flexibility was seen in vitro as well as in *S. cerevisiae*, especially for the last nine amino acids [21]. Still, it is conceivable that this is not the case for *A. niger*. Alternatively it might be that in case of the non-functional P2A, proteases from *A. niger* cleave the fusion protein. *A. niger* is well-known for high-level protease production expression, even in the recipient strain used in this study, which is deficient in a transcriptional regulator reducing the expression of extracellular proteases [22]. Therefore, it might be possible that detection of unprocessed protein is not possible because endogenous proteases cleave them. To analyse this, a different antibody would have to be used. Antibodies for ketoisovalerate reductase or enniatin synthetase are not commercially available, the only option would be the use of a 2A antibody. However, no controls expressing the genes monocistronically can then be used, unless they are tagged with the required amino acids.

## Conclusions

In this study, we proved that the P2A-mediated polycistronic gene expression is possible in *A. niger*. We were interested in whether or not the position of a gene within this construct matters and therefore expressed the reporter gene luciferase at position one, two and three. In addition, two genes required for enniatin production were expressed. We were able to show that position indeed matters for luciferase, as its activity was lowest at position one. We could furthermore demonstrate that the secondary metabolite enniatin can be produced polycistronically which provides proof-of-concept that polycistronic secondary metabolite biosynthesis is possible in *A. niger*.

## Methods

### Molecular methods

The 2A peptide published in 2011 by Kim et al. was codon optimised for *A. niger* using the website http://www.kazusa.or.jp/codon/ and ordered as primer dimers [14, 23]. Genes of interest (*luc*, *esyn*, *ekivR*) were amplified with the Q5 proof-reading polymerase according to the manufacturer instructions using primers with 15–18 overlapping base pair regions. Plasmids were constructed using the Gibson assembly reaction [24]. Details on the reaction mix are described in reference [18]. For transformation of *Escherichia coli* TOP10 cells 25 μl of competent cells were mixed with 3 μl of the Gibson reaction. Ampicillin at a concentration of 50 μg/ml was used for selection. Plasmids were analysed for correct assembly by restriction analysis and sequencing of overlapping base pair regions of the assembled fragments. In addition, PCR products containing the P2A fragments amplified from strain TS39.4 were analysed by sequencing to prove presence of the alanine coding codon. All primers used and details on the assembly can be found in Additional file 1. All polycistronic expression cassettes were transformed into the *pyrG* $^-$ recipient strain AB1.13 [22] and transformants were screened based on uridine prototrophy. *A. niger* transformation, selection procedures and fungal chromosomal DNA isolation were done according to Arentshorst et al. [25]. RNA was isolated from freeze dried biomass (24 h grown in complete medium with 20 μg/ml doxycycline) using TRIzol^™^ (Thermo Fisher). Southern and Northern analysis was done on a nylon membrane (Roth) using DIG labelled probes and CDP-Star^®^ (Sigma-Aldrich) as a substrate. The Riboruler High range (Thermo Fisher) was used as RNA ladder.

### Strains and cultivation conditions

*Escherichia coli* TOP10 strains were grown in LB medium, supplemented with 1.5% agar-agar and ampicillin when necessary, at 37 °C. *A. niger* strains were grown at 30 °C using complete or minimal medium supplemented with 1 mM uridine where necessary. For details on medium composition the reader is referred to Arentshorst et al. [25]. For induction 20 μg/ml doxycycline hyclate (Sigma-Aldrich) was used. Biomass was separated from medium by vacuum filtration. Cultures grown for enniatin B determination were supplemented with 10 g/l talcum to ensure similar growth morphologies, containing 1 or 5% (w/v) glucose, which were induced 16 h post inoculation and grown at 26 °C [3]. For protein extraction samples were induced 8 h post inoculation and allowed to grow for 24 h if not specified otherwise.

### Protein extraction and analysis

Proteins were extracted from 10 mg ground freeze dried biomass using 100 μl sample loading dye diluted with 50 mM sodium phosphate buffer pH7 (4× loading dye: 40% glycerol, 0.08% bromophenol blue, 8% SDS, 4% 2-mercaptoethanol, 50 mM EDTA, 200 mM Tris-HCl pH 6.8). Samples were immediately boiled for 5 min at 95 °C. Solids were separated by centrifugation at 19,090×*g* for 5 min. Samples were run on a 5–15% (w/v) gradient SDS-PAGE. Proteins were blotted onto a PVDF membrane (Roth). The monoclonal Luci17 anti-luciferase primary antibody (Thermo Fisher, diluted 1:3000) was used. This antibody was detected with an anti-mouse-HRP conjugated antibody (Dako, diluted 1:3000). Primary and secondary antibody incubations were done in PBS-T (137 mM NaCl, 2.7 mM KCl, 1 mM Na$_2$HPO$_4$, 0.2 mM KH$_2$PO$_4$, 0.1% Tween-20) supplemented with 5% powdered milk. Incubation times were 16 h at 4 °C for the primary and 1 h at room temperature for the secondary antibody. The WesternBright kit (Biozym) was used as a substrate for the chemiluminescence reaction and signals were detected with the ChemiDoc^™^MP Imaging System (Biorad) using the Image Lab software.

### Luminescence assay in microtiter plates

Luciferase activity was determined by measuring bioluminescence in triplicate in 96 well plates. There, 200 μl of complete medium supplemented with 1 mM uridine were mixed with 2.7 μl luciferin (25 mM, Promega) and 2 μg doxycycline. Wells were inoculated with 10 μl of a 10^6^ spores/ml suspension. The luminescence in counts per second (cps) was detected by a photomultiplier tube using a Victor3^™^ Perkin Elmer plate reader. Optical density (OD) was measured at 595 nm using the same instrument.

### Enniatin B determination

The freeze dried solid fraction (biomass and talcum) was ground and approximately 25 mg were mixed with 1 ml of methanol. This mixture was incubated at room temperature for 1 h at 500 rpm. Solids were separated by centrifugation at 19,090×*g* for 15 min. Samples were analysed on a Bruker ultrafleXtremeTM equipped with a smartbeam II laser, in positive linear mode and diluted where necessary. CHCA ($\upalpha$-cyano-4-hydroxycinnamic acid, Sigma Aldrich) was used as a matrix at a concentration of 20 mg/ml in an acetonitrile-water mixture (1:1) acidified with formic acid (1%). Acetonitrile and formic acid were purchased at VWR. Deuterated enniatin, kindly provided by Lennart Richter (TU Berlin), was used as an internal standard and added to the matrix at a concentration of 5 μg/ml. One μl sample was mixed with 1 μl of matrix, applied onto the polished sample target and allowed to dry. Calibration was done using a peptide standard mixture from Bruker with the following [M+H]^+^ values: angiotensin II (1046.5418), angiotensin I (1296.6848), substance P (1347.7354), bombesin (1619.8223), ACTH clip 1–17 (2093.0862), ACTH clip 18–39 (2465.1983), somatostatin 28 (3147.4710). Spectra were recorded in a mass range of 500–3000 Da. The laser was used at a 50% intensity, with a frequency of 2 kHz. 100–200 shots were used to ionise the matrix-sample mixture. Analysis was done using the flex Analysis 3.4 software from Bruker Daltonik GmbH. The concentration of enniatin B ($c_{ennB}$) was calculated using the following formula and automated integration of the sodium adduct of enniatin B (ennB) as well as the deuterated internal standard (deut-ennB):

$$\begin{aligned} c_{ennB} = \frac{{A([M+Na]}^{+}_{ennB})}{{A([M+Na]}^{+}_{deut-ennB})}\cdot c_{deut-ennB}. \end{aligned}$$

## Additional file

**Additional file 1.** Primers used in this study including cloning strategies and Southern blots.
