# Supplementary material for: Polycistronic gene expression in Aspergillus niger
Source: Microb Cell Fact. 2017 Sep 25;16:162. doi: 10.1186/s12934-017-0780-z (PMC5613464; doi:10.1186/s12934-017-0780-z)
Supplement: Supplementary file 1 — Additional file 1. Primers used in this study including cloning strategies and Southern blots. [file 12934_2017_780_MOESM1_ESM.pdf]

1 Primers and Plasmids

Table 1: Primers used in this study, linker sequences are indicated in lower case

Primer name	Primer sequence
255	TGCTTAATCAGTGAGGCACC
432	AGTAGATGCCGACCGGGA
560	ccggtcggcatctactgtttaaacttaGAGCTTGGACTTGCCGCCCT
632	GGCTCCGGCGCCACCAACTTCTCCCTCCTGAAGCAGGCCGGTGAC
633	GGGGCCGGGGTTCTCCTCGACGTCACCGGCCTGCTTCAGGAGGGA
635	ATGGAAAAGCCTGTGGCCTCA
636	TAATGTCCTCCTAGCCAACTCATCC
637	ATGTCACTCCACACCCCAAG
638	CAAAGCCTCGTTCAAACCATTG
639	ATGGAGGACGCCAAGAACA
640	GAGCTTGGACTTGCCGCC
641	ggcggcaagtccaagctcGGCTCCGGCGCCACC
642	gttcttggcgtcctccatGGGGCCGGGGTTCTCC
643	ttggctaggaggacattaGGCTCCGGCGCCACC
644	ggccacaggcttttccatGGGGCCGGGGTTCTCC
645	ggccacaggcttttccatGGCGCCGGGGTTCTCC
646	ggtttgaacgaggctttgGGCTCCGGCGCCACC
647	tggggtgtggagtgacatGGGGCCGGGGTTCTCC
648	tggggtgtggagtgacatGGCGCCGGGGTTCTCC
649	ttgagcagacatcaccgtttaaacaccATGGAAAAGCCTGTGGCCTCA
652	ccggtcggcatctactgtttaaacttaCAAAGCCTCGTTCAAACCATTG
720	tgaggttttgaacaaGGATGAGTTGGCTAGGAGGAC
762	cgcaggttggatctgctggaAATCAAGAGGACTAACTACC
763	GGTAGTTAGTCCTCTTGATTTCCAG
764	cagatcgctgagataGGTGCCTCACTGATTAAGCA
778	TACAGCTGCGTTTGCG
779	CATCGACAAGGACGGC

Table 2: Plasmids used in this study, * refers to a mutated P2A

Plasmid name	Description	Reference
pXM1.1	P_{gpdA}::rtTA2^S-M2::T_{cgrA}::*tetO7*::P_{min}::*luc*::T_{trpC}	Wanka *et al.* 2016
pÖV7.3	P_{gpdA}::rtTA2^S-M2::T_{cgrA}::*tetO7*::P_{min}::*ekivR*::T_{trpC}	not published
pDS4.2	P_{gpdA}::rtTA2^S-M2::T_{cgrA}::*tetO7*::P_{min}::*esyn1*::T_{trpC}	Richter *et al.* 2014
pVG2.2	P_{gpdA}::rtTA2^S-M2::T_{cgrA}::*tetO7*::P_{min}::T_{trpC}	Meyer *et al.* 2011
pTS32.372	pVG2.2 with *luc-P2A-ekivR-P2A-esyn1*	this study
pTS35.41	pVG2.2 with *luc-P2A-ekivR*	this study
pTS36.6	pVG2.2 with *luc-P2A**-*ekivR-P2A**-*esyn1*	this study
pTS37.13	pVG2.2 with *ekivR-P2A-luc-P2A-esyn1*	this study
pTS38.3	pVG2.2 with *ekivR-P2A-esyn1-P2A-luc*	this study

2 Plasmid assembly

The P2A peptide was generated by fusing primer 632 and 633.
P2A sequence: GGCTCCGGCGCCACCAACTTCTCCCTCCTGAAGCAGGCCGGTGACGTCGAGGAGAAC-CCCGGCCCC. The corresponding overlapping base pairs needed for Gibson assembly were introduced by primers. All PCRs for the genes and P2A fragments with the corresponding linkers are shown in Table 3.
All genes were expressed from the Tet-On system. The plasmid pVG2.2 linearised with PmeI was used as

a backbone. Assembly of a plasmid containing the *esyn1* gene when building pTS32.* failed. Therefore, the *esyn1* gene was divided in two fragments.

Table 3: PCR reactions for building blocks

PCR #	Primer pair used	5' overlap	3'overlap	Template for PCR	Short description
1	635&636			pÖV7.3	*ekivR* ORF no stop
2	637&638			pDS4.2	*esyn1* ORF no stop
3	639&640			pXM1.1	*luc* ORF no stop
4	641&644	*luc*	*ekivR*	P2A	functional P2A with overlap to *luc* and *ekivR*
5	643&647	*ekivR*	*esyn1*	P2A	functional P2A with overlap to *ekivR* and *esyn1*
6	641&645	*luc*	*ekivR*	P2A	non-functional P2A with overlap to luc and *ekivR*
7	635&648	*ekivR*	*esyn1*	P2A	non-functional P2A with overlap to *ekivR* and *esyn1*
8	641&647	*luc*	*esyn1*	P2A	functional P2A with overlap to *luc* and *esyn1*
9	646&644	*esyn1*	*ekivR*	P2A	functional P2A with overlap to *esyn1* and *ekivR*
10	643&642	*ekivR*	*luc*	P2A	functional P2A with overlap to *ekivR* and *luc*
11	646&642	*esyn1*	*luc*	P2A	functional P2A with overlap to *esyn1* and *luc*
12	764&640	*ampR*		pTS35.41	fragment from *ampR* to *luc* including Tet-On

Table 4: Assembly of pTS32.372

PCR #	Primer pair used	5' overlap	3'overlap	Template for PCR	Short description
13	432&255	T_{trpC}	*ampR*	pVG2.2	fragment from T_{trpC} to *ampR* including *pyrG**
14	764&636	*ampR*	*ekivR*	pTS35.41	fragment from *ampR* to *ekivR*, including Tet-On, *luc*
15	720&763	*ekivR*	esyn1/2	fusion of #5 and pDS4.2	first half of *esyn1*
16	762&652	esyn1/2	T_{trpC}	pDS4.2	second half esyn1

Table 5: Assembly of pTS36.6

PCR #	Primer pair used	5' overlap	3'overlap	Template for PCR	Short description
17	432&255	T_{trpC}	*ampR*	pVG2.2	fragment from T_{trpC} to ampR including *pyrG**
18	764&645	*ampR*	*ekivR*	fusion of #12 and #6	fragment from *ampR* to *luc* including Tet-On
19	635&763	*ekivR*	esyn1/2<br>with non-functional P2A	fusion of #6 & pDS4.2	fragment from *ekivR* to half *esyn1*
20	762&652	esyn1/2	T_{trpC}	pDS4.2	second half of *esyn1*

Table 6: Assembly of pTS37.13

PCR #	Primer pair used	5' overlap	3'overlap	Template for PCR	Short description
21	432&255	T_{trpC}	*ampR*	pVG2.2	fragment from T_{trpC} to *ampR* including *pyrG**
22	764&642	*ampR*	*luc*	pTS38.3	fragment from *ampR* to *ekivR*
23	639&647	*luc*	*esyn*	pTS35.41	*luc* gene with linkers
24	637&763	*esyn*	esyn1/2	pDS4.2	first half of *esyn1*
25	762&652	esyn1/2	T_{trpC}	pDS4.2	second half of *esyn1*

Table 7: Assembly of pTS38.3

PCR #	Primer pair used	5' overlap	3'overlap	Template for PCR	Short description
26	649&763	P_{min}	esyn1/2	pTS32.372	first half of *esyn1*
27	762&642	esyn1/2	*luc*	fusion of #2 and #11	second half of *esyn1*
28	639&560	*luc*	T_{trpC}	pXM1.1	*luc* gene with linkers

3 Plasmid maps

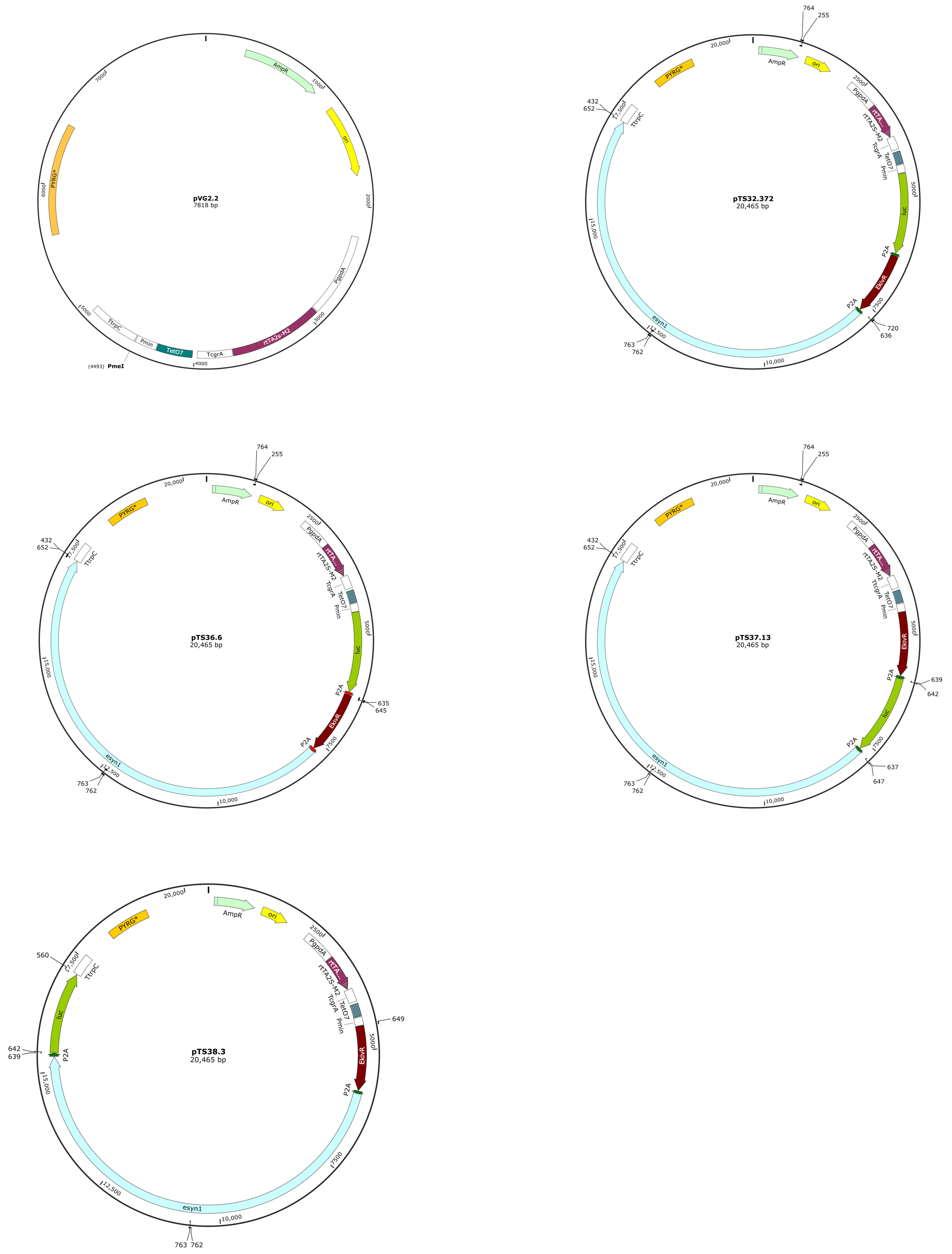

Figure 1: Plasmid maps of assembled plasmids and the entry vector pVG2.2 highlighting the restriction site PmeI. The primers used for the final assembly of the building blocks are shown. Plasmid maps were generated using SnapGene Viewer and modified with Inkscape.

4 Southern Analyses

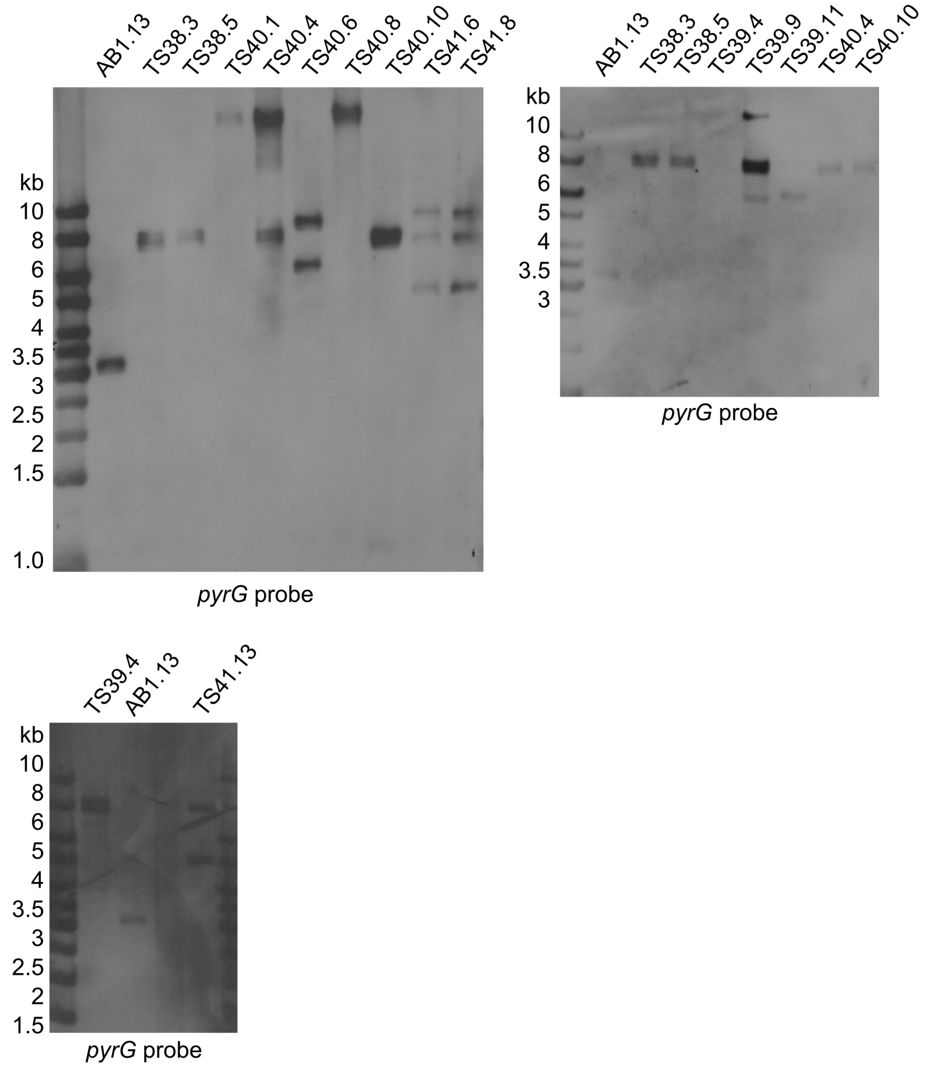

Figure 2: Southern analysis of strains polycistronically expressing luciferase, ketoisovalerate reductase and enniatin synthetase. Using the *pyrG* probe and NcoI for restriction, the recipient strain will give a signal at 3.1 kb. The strains TS38, TS39, and TS40 will give a signal at 7.7 and 8.1 kb in case of single integration, for tandem integration an additional 12.7 kb signal. For strain TS41 a signal at 7.7 and 5 kb is expected in case of single integration, for tandem integration an additional signal at 9.6 kb.
